# Stage-Specific Histone Modification Profiles Reveal Global Transitions in the Xenopus Embryonic Epigenome

**DOI:** 10.1371/journal.pone.0022548

**Published:** 2011-07-22

**Authors:** Tobias D. Schneider, Jose M. Arteaga-Salas, Edith Mentele, Robert David, Dario Nicetto, Axel Imhof, Ralph A. W. Rupp

**Affiliations:** 1 Department of Molecular Biology, Adolf-Butenandt Institut, Ludwig-Maximilians-Universität München, Munich, Germany; 2 Medizinische Klinik und Poliklinik I, Klinikum Grosshadern, Ludwig-Maximilians-Universität München, Munich, Germany; 3 Center for Integrated Protein Science Munich, Munich, Germany; University of Birmingham, United Kingdom

## Abstract

Vertebrate embryos are derived from a transitory pool of pluripotent cells. By the process of embryonic induction, these precursor cells are assigned to specific fates and differentiation programs. Histone post-translational modifications are thought to play a key role in the establishment and maintenance of stable gene expression patterns underlying these processes. While on gene level histone modifications are known to change during differentiation, very little is known about the quantitative fluctuations in bulk histone modifications during development. To investigate this issue we analysed histones isolated from four different developmental stages of *Xenopus laevis* by mass spectrometry. *In toto*, we quantified 59 modification states on core histones H3 and H4 from blastula to tadpole stages. During this developmental period, we observed in general an increase in the unmodified states, and a shift from histone modifications associated with transcriptional activity to transcriptionally repressive histone marks. We also compared these naturally occurring patterns with the histone modifications of murine ES cells, detecting large differences in the methylation patterns of histone H3 lysines 27 and 36 between pluripotent ES cells and pluripotent cells from Xenopus blastulae. By combining all detected modification transitions we could cluster their patterns according to their embryonic origin, defining specific histone modification profiles (HMPs) for each developmental stage. To our knowledge, this data set represents the first compendium of covalent histone modifications and their quantitative flux during normogenesis in a vertebrate model organism. The HMPs indicate a stepwise maturation of the embryonic epigenome, which may be causal to the progressing restriction of cellular potency during development.

## Introduction

The packaging of DNA into chromatin plays an important role in setting up specific gene expression patterns during early embryonic development. The level of packaging is regulated by specific histone modifications leading to a more open or closed chromatin structure, depending on the factors that bind to the specific modifications. Recently, several genome wide mapping studies have revealed specific distribution patterns of particular modifications [1]. For example, H3 Lys-4 (i.e. H3K4) is often found methylated at cis-regulatory elements such as enhancers and promoters, and a combinaton of di-/trimethylated K4 at the promoter and K36 in the transcribed gene body, respectively, indicates reliably active transcription units. In contrast, methylations at H3K9 and H3K27 correlate with repressed domains. Under specific circumstances, chromatin fragments have been identified that simultanously carry active as well as repressive modifications, specifically the H3K4me3 and H3K27me3 marks. This chromatin state has been termed bivalent to indicate its ambiguous nature [2], [3], [4], [5]. Bivalent domains first have been described in embryonic stem (ES) cells, where they may keep developmental regulatory genes in a state poised for subsequent activation during cellular differentiation. However, bivalent domains are not restricted to pluripotent cells and most likely have functions beyond priming genes for activation [6]. Altogether, these genome wide studies indicate covalent histone modifications as important regulatory components of development and cellular differentiation.

A growing body of data suggests that histone modifications convey important information to early developmental programs in mammals. Histone modification patterns in gametes become established during germ cell differentiation [7], and nucleosomes, which are retained in low numbers in human sperm, carry histone modifications on particular developmental loci [8]. This finding extends to zebrafish, who packages its sperm genome entirely into nucleosomes, rather than protamines [9]. After fertilisation, widespread epigenetic reprogramming, occuring in cooperation with stochastic gene expression of lineage-determining transcription factors, helps prepare the earliest lineage decisions that generate trophectoderm, primitive endoderm and inner cell mass (reviewed in detail in refs. [10], [11], [12]).

Our knowledge about the epigenetic changes, which accompany the determination of definitive somatic cell lineages in the mammalian embryo, are derived largely from in vitro differentiation studies on pluripotent ES cells. Generally, ES cells are characterized by a derepressed chromatin structure that is highly permissive to the transcriptional machinery [13]. The chromatin structure and function of ES cells changes dramatically during differentiation. For instance, the number and size of heterochromatic foci, visualized by immunostaining for heterochromatin protein 1 (HP1), increase upon differentiation [14]. FRAP (fluorescent recovery after photo-bleaching)-analysis revealed that HP1 as well as core and linker histone proteins are hyper-dynamically bound to chromatin in the undifferentiated ES cell, but are more stably associated with chromatin upon differentiation. These changes extend also to covalent histone modifications. Examples are the differentiation-dependent increase in the repressive mark tri-methylated Lys-9 of histone H3 (H3K9me3), and the observed decrease in the global levels of acetylated histones H3 and H4 [14]. ES cells, differentiated under retinoic acid administration, undergo a two-fold reduction of H3K4me2 levels, while H3K9me1 and H3K79me2 levels increased moderately, describing nearly specific histone modification patterns for undifferentiated ES cells and their differentiated progeny [15]. Striking changes in histone methylation and acetylation during human ES cell differentiation have also been observed by mass spectrometry for the aminoterminal tail domain of histone H4 [16]. Overall, this data shows that the fraction of nucleosomes with repressive histone marks increases during ES cell differentiation, reflecting the lineage-specific use of the genome in somatic cells. Interestingly, direct reprogramming of somatic cells to an induced pluripotent state reverses these changes largely, although not completely. During reprogramming, histones become hypermethylated at H3K4, bivalent domains are re-established, while repressive marks are diminished, indicating a general shift towards the transcriptionally permissive chromatin of pluripotent ES cells [17]. Thus, transitions between pluripotent and comitted somatic cell fates during development or reprogramming involve global alterations in histone-modifications.

Pluripotent ES cell lines are established and maintained under stringent culture conditions. Although being derived from endogenous pluripotent cell populations, they have no strict counterpart *in vivo* [12]. Indeed, derivation of ES cells from the blastocyst inner cell mass causes a significant skewing of histone modification patterns, including H3K4 and H3K27 methyl marks, compared to the inner cell mass [18]. The many cell types and tissues of the mammalian embryo arise from the epiblast after implantation, and very little is known about histone modifications at this time of development. Results from carrier ChIP analysis on microdissected tissues from post-implantation mouse embryos suggest that genes known to be bivalently marked in ES cells tend also to be enriched for these modifications in embryonic tissues [19]. However, the bivalent status of these genes has not been rigorously established by sequential ChIP analysis and, thus, the opposing marks could be distributed between cells in a non-overlapping manner.

Other vertebrate model organisms such as *Xenopus* and zebrafish have recently provided key information on epigenetic changes in early development. Histone variants and modifications from various *X. laevis* cell types including oocytes, sperm and somatic cells, have been characterized by immunoblotting and mass spectrometry, indicating unique histone modification signatures for each cell type [20], [21]. While these studies did not analyse chromatin from normal embryos, genome-wide RNA-Seq and ChIP-Seq technologies were combined to investigate histone modifications in the course of *X. tropicalis* development [22]. This revealed a hierarchical acquisition of H3K4me3 and H3K27me3 marks, following the onset of zygotic transcription at midblastula. Specifically, spatial differences in the deposition of the repressive Polycomb mark H3K27me3 was predicitve of localized gene expression patterns. In contrast to mammalian ES cells, bivalent chromatin domains are practically absent from Xenopus embryos [22]. Lysine trimethylation of H3K4 and H3K27 appears in the zebrafish epigenome only after the maternal-zygotic transition and in the same sequence as in frogs. However, sequential chromatin immunoprecipitation revealed the existence of bivalent chromatin domains in the zebrafish [23]. While it is currently unclear, whether the observed differences for bivalent domains between frog and fish has a biological basis or reflects technological differences, these studies have provided evidence of genome-wide transitions in histone modifications during normal vertebrate development.

The information from ChIP-based studies is invariably dictated by many technical parameters, most notably the antibody quality (discussed in [24]). We have sought to obtain quantitative information on different histone modifications by an antibody-independent approach. Here we report the results from mass spectrometry analysis of histone modifications present in bulk embryonic chromatin through *X. laevis* development. This analysis has revealed major quantitative shifts for several histone modifications known to be involved in gene regulation, and has also identified specific differences between pluripotent cells from frog embryos and murine ES cells. The quantitative shifts, which we observed during development, cluster into stage-specific histone modification profiles accompanying and potentially regulating the transition from pluripotent to determined cell states.

## Results

### Experimental design

**Figure 1:**
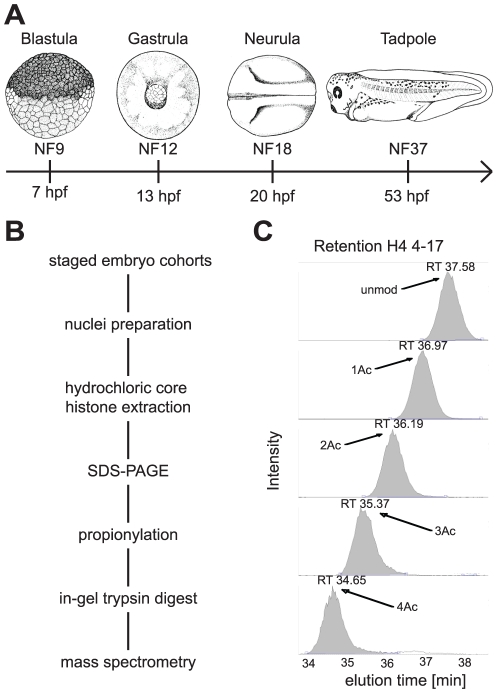
Histone modification profiling for *Xenopus* embryogenesis. **A**) Time line of *Xenopus laevis* embryonic development – NF stages according to Nieuwkoop and Faber [25] and by hours after fertilization (hpf). The stages selected for mass spec analysis are characterized by the following embryonic and cellular features: Blastula (NF9) naivé/multipotent cells; Gastrula (NF12) germ layers specified; Neurula (NF18) germ layer patterning and differentiation; Tadpole (NF37) embryonic development completed, larvae hatched. **B**) Flow chart for quantitative mass spectrometric analysis of histone modifications from normal *X. leavis* embryos. **C**) Elution profile of differentially acetylated histone H4 4-17 peptides of *X. laevis* Blastulae from a reversed-phase C18 micro-column of the LC-MS/MS. Shown are the extracted ion chromatograms at corresponding retention times (x-axis), the y-axis represents the intensity of ion currents of the quadrupole Orbi-Trap mass spectrometer. The areas under the peaks are used for quantification of different modification states of a peptide. X-axis: elution time in minutes. Y-axis: intensity of peptides according to ion current of the quadrupole Mass Spectrometer (unmod  =  unmodified peptide, 1Ac  =  monoacetylated, 2Ac  =  diacetylated, 3Ac  =  triacetylated, 4Ac  =  quatruple-acetylated peptides, RT  =  retention time).

In order to investigate histone post-translational modifications (PTM) during vertebrate embryogenesis, we purified core histone proteins from unmanipulated *X. laevis* embryos of four different developmental stages (Figure 1A). The stages included blastula (Nieuwkoop Faber stage NF9), gastrula (NF12), neurula (NF18) and tadpole (NF37) embryos [25], representing key steps in vertebrate development. At late blastula, shortly after the onset of zygotic transcription, embryos consist mostly of uncomitted, pluripotent cells (refs. [26], [27]; Nicetto and Rupp, unpublished data). By the gastrula stage, the germ layers have been induced, and embryonic patterning increases the cellular diversity of the embryos during neurulation [28], [29]. After hatching, tadpoles are composed largely of differentiated, although premetamorphic, somatic cells [25].

To evaluate the results from the embryonic samples, we also prepared histones from Xenopus A6 and XTC-2 cell lines, as well as from murine germline-transmission competent GS-1 ES cells [30], [31] and primary embryonic fibroblasts (MEF). The identity of the histone molecules was determined by mass spectrometry. Alignment of the primary sequences of *Xenopus* histones with core histones of other species such as human, mouse and fruitfly revealed high interspecies conservation of the H3 and H4 histones (Figure S1). Overall, we obtained a sequence coverage of 68.9% for H3 and 87.3% for H4 (Figure S2 and data not shown), covering most of the known histone modification sites on these histones [32], [33]. Therefore, we focused our further investigations on canonical, replication-dependent histone H3 and on histone H4.

### Histone Modification Analysis and Quantification using LC-MS/MS

For the identification of histone post-translational modifications we preferentially used high mass accuracy LC-MS/MS mass spectrometry. To obtain peptides of suitable masses, histones H3 and H4 were separated by SDS-PAGE, covalently modified by propionic anhydride and subsequently digested by the endoprotease trypsin [34] (see Figure 1B). The resulting peptides were then separated by C18 reversed phase chromatography, subjected to tandem mass spectrometry and quantified by integrating the area under the extracted ion chromatogram (XIC) of the corresponding doubly and triply charged ions. When we analysed the chromatographic behaviour of differentially modified peptides, we detected a specific shift of retention times depending on the type of modification. Specifically, acetylation of the H4 peptide amino acids 4-17 leads to an earlier elution by approximately 0.7 minutes (Figure 1C). From these findings we concluded that in addition to the mass of a histone peptide and its fragmentation spectrum, the elution time is also a unique parameter to characterise the modification of a peptide. In the following analysis we therefore used the retention time as a third parameter to determine the identity of a given peptide. The small mass and specific biochemical characteristics of two peptides, i.e. histones H3 peptide 3–8 and H4 peptide 20–23, precluded analysis by LC-MS/MS, and, therefore, were analysed by conventional MALDI-TOF mass spectrometry to investigate their modification states.

Overall, we monitored 18 known modification sites (17 lysine residues, one serine residue) that are present on five peptides of H3 and four peptides of H4, respectively (Tables S1 and S2). Quite often, the type or extent of modification on a particular site is known to be associated with different biological functions. For instance, the Lysine residue at position 27 of H3 can be unmodified or acetylated, which is compatible with transcriptional activity; alternatively, it can be mono-, di- or trimethylated, indicating an increasing activity by Polycomb, which leads to transcriptional repression. Furthermore, the fragmentation of propionylated histones generates in some cases peptides, which contain more than one modification site (e.g. peptide 4–17 on H4; see Tables S1, S2, S3 and S4), allowing us to detect some combinatorial modification states that coexist on single peptide molecules. Based on the potential biological implications we view each unambigously identified mass shift as a distinct modification state. Based on this definition, we have characterized in toto 59 different modification states by either LC-MS/MS (52 states; Tables S3 and S4) or by MALDI/TOF (7 states). For each developmental stage we quantified spectra from two (tandem LC-MS/MS) or three (MALDI-TOF) biological replicates, with an average correlation coefficient of 0.9962 between biological replicates, indicating high robustness for the reported epigenetic states.

### Active marks

**Figure 2:**
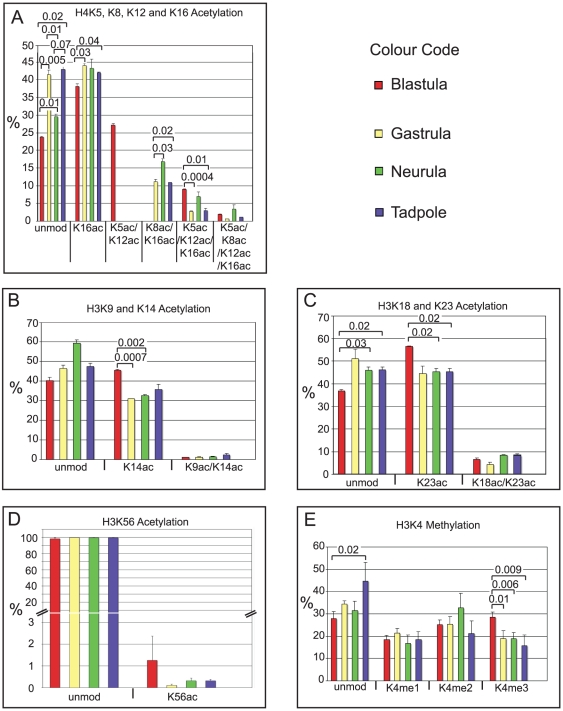
Histone modifications of cell proliferation and active transcription in *Xenopus* embryos. Bar-Charts showing the relative abundance of histone modifications, identified either by Orbi-Trap LC-MS/MS mass spectrometry (**A-D**; error bars indicate SD of two independent biological replicates) or MALDI-TOF mass spectrometry (**E** - error bars indicate SD of three independent biological replicates). Abbreviations: unmod  =  unmodified peptide, Kac  =  acetylated lysine residue, Kme1  =  mono-methylated, Kme2  =  di-methylated, Kme3  =  tri-methylated lysine residue. Where applicable, p values are given by numbers above brackets to indicate significant differences in the abundance of a histone modification between samples.

As described above, we detected multiple acetylation states at the aminoterminal tail of histone H4 (Figures 1C and 2B). Similar to what has been described in other organisms [35], [36], the mono-acetylated peptides are predominantly modified at Lysine 16 (Figure 2A). In blastulae, 38% of the total H4 4–17 peptides carry a single acetyl group. The level of mono-acetylation rises in later developmental stages to a steady state of more than 40% abundance (see also Table S2). The percentage of mono-acetylation is slightly lower in tissue culture cells (38% in A6 and 34% in XTC-2, respectively; Figure S3, panel A), which instead contain more unmodified peptide (54% in A6 and 58% in XTC-2). Of the many potential di-acetylated forms we detected only two states, i.e. simultanous acetylation of either K5 and K12, or K8 and K16. K5/K12 di-acetylation is quite abundant at the blastula stage (27%, Figure 2A). Since this modification state is known as a hallmark of predeposition histones [35], [37], [38], this pool probably reflects maternally stored H4 molecules. This modification state was neither detected on histones from later stages nor on histones isolated from frog cell lines. From gastrula stage onwards, we detected a wave of K8/K16 diacetylated H4 in the embryo, increasing from 11% at NF12 to 17% at NF18, before decreasing again to 11% at NF37 (Figure 2A). The frog cell lines contain somewhat lower levels of K8/K16 di-acetylated H4 (appr. 7%, Figure S3A). The tri- as well as the tetra-acetylated isoforms are less abundant in all embryonic stages (<10%), even though they were 5-fold higher than in tissue culture lines (<1.5%; compare Figure 2A and Figure S3A). In case of the tri-acetylated isoform, all LC-MS/MS spectra point to a specific modification pattern of acetylation marks at lysines 5, 12 and 16, which argues against a zipper model of lysine acetylation that had been suggested previously for mammalian histones [39].

On the H3 N-terminus, we find acetylation of K9 and K14. At low abundance we detected di-acetylated peptides (K9ac + K14ac) in all stages, however, we were not able to find acetylation of K9 without the acetylation of K14 (Figure 2B). This suggests that K9 acetylation either requires previous acetylation of K14 or occurs simultanously. The level of mono-acetylation of this peptide decreases significantly as development proceeds from 45% in blastulae to 31% in gastrulae, after which it increases again gradually up to 37% in tadpoles. As it is the case for H4 mono-acetylation, the tissue culture cells contain less acetylated H3 (Figure S3 panel B). Di-acetylated H3 K9/K14 molecules are much less abundant and their levels do not change significantly during development. However, the levels are slightly lower again in the two frog cell lines, compared to blastulae, which contain the lowest value of the four embryonic stages (1.17%, see Table S1).

In addition to the K9 and K14 residues, the nearby lysines 18 and 23 on the H3 N-terminal tail can also be acetylated. We find a similar pattern for both mono-acetylated states with a significant shift between NF9 and the three other embryonic stages (Figure 2C). Like for the K9/K14 pair, we find K18 acetylation only in combination with acetylated K23. These levels are between 4% in NF12 and 9% in NF37. Monoacetylated K23 ranges from 57% in Blastula to 45% in the other embryonic stages. In the two *Xenopus* cell lines, the level of K23 mono-acetylation is around 20% and the di-acetylation at 2% (Figure S3C). Thus, also for these H3 acetylation marks, the four embryonic samples are very different from the cultured frog cell lines.

Further inwards of the N-terminal tail, we also detected acetylation of K27. This Lysine can also be methylated by PRC2 complexes (see below). Embryonic samples contain H3K27ac at relatively constant levels of 1–2% (Table S1). This modification was less abundant in all other samples (≤0.54% (Figure S3, panel I), and among these, ES cells had the lowest K27ac levels (0.06%; Table S1). These differences, in particular between frog embryos and murine ES cells, are remarkable, given that acetylation of K27 is known to antagonize Polycomb silencing in flies [40], and to mark active enhancers [41].

Besides N-terminal acetylation marks, we also detected acetylation in the H3 histone-fold domain at Lysine 56 (H3K56ac; Figure 2D). This modification has been suggested to represent a predeposition mark [42], [43], [44], and it was shown to be linked to the core transcriptional network of human ES cells [45]. In mammalian cells increased H3K56ac levels correlate with tumorigenicity and the undifferentiated nature of cells [46]. In frog embryos, H3K56ac levels are always low (0,11%–0,32% abundance) and comparable to the levels in the two frog cell lines.

We have also found Lysine-56 to be methylated (predominantly me1) in both embryos and cultured cell lines at low abundance (Table S1). Very little is known about this PTM, which has been detected so far in human and *Tetrahymena* chromatin [47]. Interestingly, both K56me1 and K56ac levels reach a minimum at gastrula stages, i.e. when germ layers become specified. MEFs and murine ES cells contain somewhat higher levels of K56me1 (Table S1). Since this mark has also been observed in *Drosphila* embryos (A.I., unpublished), the ability to methylate H3K56 appears to be a conserved feature among metazoa.

The most prominent histone modification associated with transcriptionally active promotors is di- and tri-methylated lysine 4 of H3 (H3K4me2/me3) [1], [48]. In order to analyse the tryptic peptide 3-8 that contains Lys-4, we used a different de-salting method that can be applied to very small hydrophilic peptides. This new method improves the signal to noise ratio significantly, thereby allowing the quantitation of the differentially modified isoforms (Figure 2E). We found that the amount of the unmodified peptide increases significantly during embryonic development from 27% in NF9 to 44% in NF37. This is largely due to a decrease in tri-methylation from 28% in NF9 to 16% in NF37. The levels of the mono- and di-methylated isoforms remain relatively constant. In comparison, the two cultured frog cell lines have much higher levels of unmodified (67% in A6 and 62% in XTC-2), and much lower levels of di- and tri-methylated H3K4 (≤10% each; Figure S3, panel F). This data indicates an unusual and transcriptionally permissive state of the uncomitted blastula epigenome, in which half of the histone H3 N-terminal tails appear to be di- or tri-methylated at Lysine 4.

### Repressive marks

**Figure 3:**
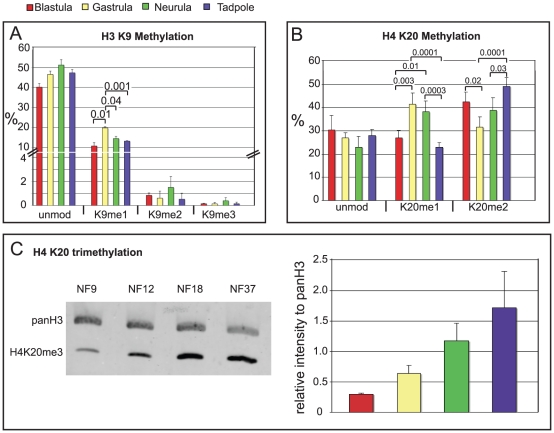
Histone Modifications of transcriptionally repressed chromatin and constitutive heterochromatin in *Xenopus* embryos. Bar-Charts of repressive lysine methylation states on histone H3K9 and H4K20. **A**) Data from Orbi-Trap mass spectrometry - error bars: SD of two independent biological replicates. **B**) Data from MALDI-TOF mass spectrometry - error bars: SD of three independent biological replicates. **C**) Immunoblotting for total Histone H3 and histone H4 trimethylated at lysine 20. Left panel – western blot Odyssey infrared imaging signals; right panel – bar chart showing increase of H4K20me3 levels relative to total histone H3 protein during development. Abbreviations: unmod  =  unmodified peptide, Kac  =  acetylated lysine residue, Kme1  =  mono-methylated lysine residue, Kme2  =  di-methylated lysine residue, Kme3  =  tri-methylated lysine residue. Where applicable, p values are given by numbers above brackets to indicate significant differences in the abundance of a histone modification between samples.

One of the best characterized modifications that is associated with heterochromatin and transcriptionally repressed genic regions is di-/tri-methylation of lysine 9 on the H3 tail. In contrast, K9 mono-methylation has been associated with regulatory DNA elements and maintenance of activation potential during differentiation [1], [49]. In all our samples, we find K9 in mono-, di- and tri-methylated form, although at very different levels (Figure 3A). The different modification states of K9 fluctuate less than two-fold during frog development, except for K9me1, which shows a small but significant transient peak at gastrula, when it accounts for 20% of the total H3 9–17 peptides. A similar distribution of mono-, di- and trimethylated K9 is found also in the two cultured *Xenopus* cell lines (Figure S3G). Combined, the K9me2/me3 marks make up for less than 4% of the total H3 tails in the different *Xenopus* samples, suggesting a rather low density of this mark in both constitutive and facultative heterochromatin.

The tryptic peptide 20-23 from propionylated histone H4 with the sequence KVLR has one lysine residue at the position 20, which is known to be methylated. Its different methylation states have been linked to diverse epigenetic functions. The H4K20me2/me3 marks are correlated with the formation of pericentromeric heterochromatin, maintenance of genome integrity and transcriptional repression, while the role of H4K20me1 is not fully understood (see also review by [50]). Using MALDI-TOF, we found in all samples peptides corresponding to unmodified, mono-methylated and di-methylated states (Figure 3B). The amount of the unmodified peptide decreases slightly from blastula to tadpole stage, and is even lower in the A6 (20%) and XTC-2 (17%) cell lines. It is known that the specific properties of the tri-methylated H4 20-23 peptide make it very difficult to quantify this PTM by mass spec, and we have not reproducibly detected it in our samples. To determine the relative abundance of H4K20me3 in frog embryos, we performed Western blot analysis with antibodies against methylated H4K20 as well as pan-histone H3. This confirmed the consistent presence of the H4K20me3 mark during embryonic development (Figure 3C; see Figure S4 for cultured cells), and revealed also that its abundance increases appr. five-fold from blastula to tadpole stages.

### Opposing marks on the histone H3 27-40 peptide

**Figure 4:**
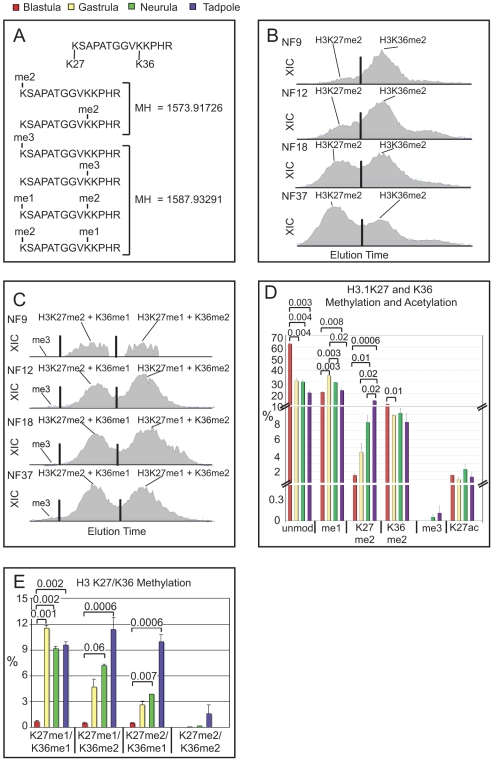
Opposing Histone Modifications on the H3 27-40 peptide in *Xenopus* embryos. **A**) Amino-acid sequence of the H3 27-40 peptide and its isobaric modification forms, which have identical mass although beeing differently modified at K27 and K36 residues. Panels **B+C** - Extracted ion chromatograms (XICs) showing separation of isobaric H3 27–40 peptides from the four Xenopus embryonic stages by differential elution from a C18 micro-column on the reversed-phase HPLC of on-line mass spectrometry. X-axis represents retention time, y-axis the intensity of ion currents in the quadrupole Orbi-Trap mass spectrometer. Peak separation was called by the ICIS peak detection algorithm program (Thermo), indicated here by the vertical black lines. **B**) XICs of isobaric di-methylated peptides, modified at either K27 or K36. **C**) XICs of isobaric tri-methylated peptides representing K27me3/K36me3, K27me2+K36me1 and K27me1+K36me2. **D**) Bar-Chart of H3K27 and K36 modification states. Note that isobaric mono- (data not shown) and tri-methylated peptides, which are methylated either at K27 or K36, elute simultanously and cannot be distinguished. **E**) Bar-Chart of combinatorial K27/K36-methylated peptides. Abbreviations: unmod  =  unmodified peptide, me1  =  single mono-methylated lysine at position 27 or 36, K27me2  =  di-methylated lysine 27, K36me2  =  di-methylated lysine 36, Kme3  =  single tri-methylated lysine at position 27 or 36, K27me1/K36me1  =  double mono-methylated, K27me2/K36me2  =  double di-methylated, K27me1/K36me2  =  combinatorial triple-methylated peptide with dimethlyted K36, K27me2/K36me1  =  combinatorial triple-methylated peptide with dimethlyted K27. Error bars represent SD. Where applicable, p values are given by numbers above brackets to indicate significant differences in the abundance of a histone modification between samples.

Methylation of lysines 27 and 36 within the histone H3 tail has been mapped to mutually exclusive regions within the genome of most eukaryotes [51]. The H3K27me2/me3 marks are almost exclusively found at inactive genomic regions, whereas the H3K36me2/me3 marks are localized predominantly in actively transcribed genes [1], [52], [53]. Both modifications reside on the same tryptic peptide (H3 residues 27–40), which allows us to determine combinations of modification states on these two lysines by mass spectrometry. However, as peptides carrying a single trimethyl group at either K27 or K36, or combinations of a monomethyl and a dimethyl group at these two lysines, are isobaric (i.e. they have the same mass), they cannot be distinguished solely by this parameter (see Figure 4A). Although tandem MS/MS analysis can facilitate the assignment of the modifications, the relative quantitation based on extracted ion chromatograms is only possible when the differentially modified isobaric peptides are physically separated. Indeed, tandem MS/MS spectra revealed that most peptides that have a higher methylation degree at position 27 elute earlier from a C18 reversed phase column than the corresponding ones that carry the same number of methyl groups on K36 (Figure 4B and C). With the exception of the isobaric mono-methylated peptides (i.e. K27me1 or K36me1), which were not separated on the C18 micro-column, this observation enabled us to identify 12 additional, distinct methylation states for the K27 and K36 positions on single H3 27–40 peptides.

In genome wide studies, tri-methylation of H3K36 is found at strongly expressed genes, which are also preferentially enriched for the replication-independent histone variant H3.3 [1], [54]. On canonical histone H3 (i.e. replication-dependent H3.2; ref. [54]) that is highly abundant in early embryos, we have detected H3K36me3 by its fragmentation spectrum only in frog tadpoles at very low abundance (see Table S3). Its relative absence from embryos could be explained, if frogs deposit the K36me3 mark selectively on the replication-independent H3.3 variant, as has been reported for murine ES cells [55]. The ratio of H3.3 to H3.2, as measured by comparing all spectra derived from the peptides unique for each isoform, decreases during development from 20% in blastula to approximately 7% in tadpoles (Figure S5). However, in all cases we find a very similar distribution of modifications within these peptides, and therefore focused our analysis on the peptide derived from the more abundant, canonical H3 molecule. Overall, this data indicates that H3K36me3 levels are low during frog development.

Consequently, these findings imply that practically all the single tri-methylated 27–40 peptides of H3 represent K27me3, a conclusion that is supported by the fragmentation spectra. We found that H3K27me3 was readily identified in many, but not all samples. Notably, it was not detected in blastula and gastrula, and appeared only at very low levels in neurula (0.05%) and tadpole stages (0.1%; Figure 4, C and D). However, in Xenopus cell lines (Figure S3, panel I), as well as in mammalian cells (see below), H3K27me3 levels were at least 100-fold higher than in bulk neurula chromatin. We conclude that the repressive K27me3 modification is present in our histone extracts, but extremely underrepresented in the early frog embryo compared to cultured frog and mammalian cell lines.

Overall, the abundance of methylated H3K27 rises during development (Figure 4D and E; Table S1). This trend became particularly obvious, when we compared the XIC profiles of the di-methylated peptide species. While the K36me2 peak stayed more or less constant, the K27me2 peak increased gradually from 1.6% in the blastula to 14% in the tadpole stage (Figure 4B and D). During this time, the ratio of H3K27me2 to H3K36me2 changed from 0.14 in the blastula to 1.6 in the neurula stage, i.e. by more than 10-fold. In comparison, the cultured cell lines A6 and XTC-2 showed the highest percentage of H3K27me2 peptides (33% and 22% respectively), and contained more than 10% of H3K27me3 (Figure S1, panel I and Table S3).

The abundance of peptides with combinatorial methylation on both K27 and K36 residues rises also siginificantly during development. In the frog blastula, less than 2% of H3 tails are simultanously modified on these sites, while one third is combinatorially modified in tadpoles (Figure 4C and E). Among these, we found also low levels of unexpected combinations such as K27me3 paired with K36me1/me2 in late embryonic and tissue culture cell samples (Table S1). In general, the level of modifications within this peptide shows a major increase during development. This trend may be connected functionally to the establishment of stable gene expression profiles as cells differentiate.

### Methylation states of pluripotent frog blastulae and murine ES cells

Most cells in the blastula stage of *Xenopus* embryos are uncomitted and capable to differentiate into derivatives of all three germ layers [26], [27]. This raised the issue, whether the histone modification profiles we observed in the frog blastula represent common features of pluripotent cells. To address this point, we continued to analyse the histone PTMs of murine ES cells and of primary embryonic fibroblasts (MEFs), which were used as feeder cells in the ES cell culture and represent a murine somatic cell type for comparison.

When we initially compared MEFs with ES cells, we observed different degrees of acetylation on the H3 18–26 and H4 4–17 peptides, indicating that we separated successfully ES cells from MEF feeders (Figure S6). In depth analysis confirmed then that the two murine cell types contained very specific profiles of histone modifications, and that the histone modifications of pluripotent cells from Xenopus blastulae were related to, but also clearly distinguishable from the profiles of pluripotent murine ES cells (Tables S1 and S2).

**Figure 5:**
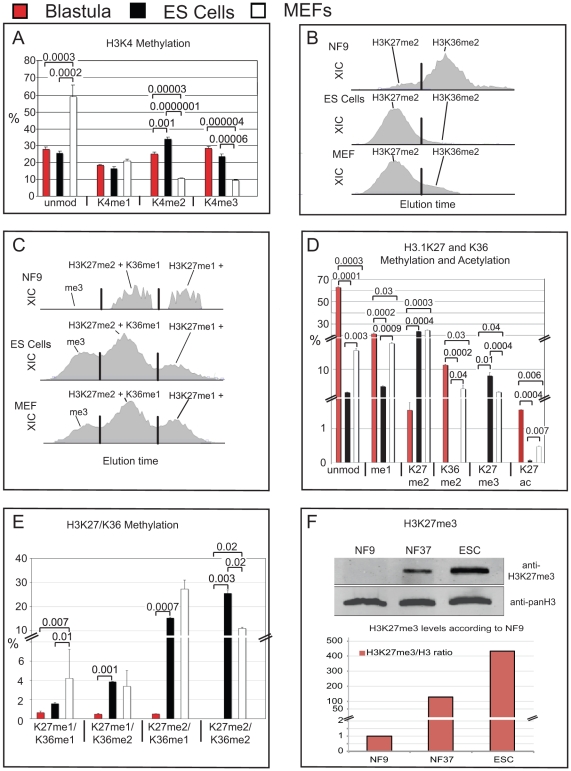
Comparison of opposing histone modifications on histone H3 from *Xenopus* blastulae, murine ES cells and MEFs. **A**) Bar-Charts showing H3K4 methylation states of *X.laevis* blastulae, GS-1 ES cells and mouse embryo fibroblasts. Data obtained by MALDI-TOF mass spectrometry, error bars indicate SD of three independent biological replicates. **B**) XIC profiles of K27me2 and K36me2 modification states of the H3 27–40 peptide of *X.laevis* blastulae, murine ES Cells and MEFs on a C18 micro-column on a reversed-phase HPLC during Orbi-Trap on-line mass spectrometry. **C**) XIC profiles for K27/K36me3, K27me2+K36me1 and K27me1+K36me2 of the H3 27–40 peptides from the same samples. In B and C, x-axis gives relative elution times of peptides, the y-axis shows their intensity according to the ion current of the quadrupole Mass Spectrometer. In B and C, X-axis represents retention time, y-axis the intensity of ion currents of the quadrupole Orbi-Trap mass spectrometer. Peak separation was called by the ICIS peak detection algorithm program (Thermo), indicated here by vertical black lines. **D**) Bar-Chart of mutually exclusive H3K27 and K36 methyl states. **E**) Bar-Chart of peptides with combinatorial H3K27 and K36 methylation. **D** and **E** - data from Orbi-Trap Mass Spectrometry, error bars indicate SEM of two independent biological replicates. **F**) Immunoblotting for total histone H3 and histone H3 trimethylated at lysine 27. Upper panel – western blot Odyssey infrared imaging signals; lower panel – bar chart showing the abundance of H3K27me3 levels relative to total histone H3 protein in Blastulae (NF9), Tadpoles (NF37) and murine GS-1 ES cells. Abbr.: unmod  =  unmodified peptide, Kme1  =  mono-methylated lysine residue, Kme2  =  di-methylated lysine residue, Kme3  =  tri-methylated lysine residue. Where applicable, p values are given by numbers above brackets to indicate significant differences in histone modifications between samples.

Bivalently marked chromatin fragments, which simultanously carry the active H3K4me3 and the repressive H3K27me3 marks, are considered a hallmark of pluripotency [3], [4]. With our technical approach we cannot determine, whether histone H3 tails are modified on both Lysine 4 and 27, since these residues are located on different tryptic peptides. Nevertheless, we compared the overall abundance of modifications at these sites. For H3K4, this comparison revealed a high similarity between Xenopus blastula and murine ES cells (Figure 5A). Of all samples, these two contained the lowest amount of unmodified K4 (below 30%), but maximal amounts of H3K4me2/me3 (>50% together). In contrast, H3K4me1 levels were not only similar between blastula, ES cells and MEFs, but almost constant in all samples (Figures 2D and S3E).

In contrast to H3K4, the methylation profiles of the H3 peptide 27–40, including Lysines 27 and 36, were very different between blastulae and ES cells. As described earlier, the majority of the di-methyl marks reside on K36 in frog blastulae. In ES cells, however, they were found predominantly on K27 (Figure 5B and D). MEFs had a similar dimethyl-distribution as ES cells, but contained significantly more K36me2 than these. A dichotomy was also found for the higher modification states of this peptide, including three to five methyl groups. Whereas in frog blastulae H3K27me3 is below the detection limit, mouse ES cells had with 8.3% the third-highest K27me3 abundance of all samples tested, and twice as much as MEFs (Figure 5C and D). This huge quantitative difference is also seen in western blots (Figure 5E). Thus, both mass spec and western blots indicate independently that the epigenome of ES cells is more than 100-fold enriched for H3K27me3 compared to frog blastulae.

Combinatorial tri-methylated states, such as K27me1/K36me2 and K27me2/K36me1 were also significantly more abundant in ES cells than in frog blastulae, and comparable to the levels found in MEFs. Notably, the highest levels of tetra- (K27me2+K36me2) and penta-methylated (K27me3+K36me2) states of the H3 27–40 petides were also found in ES cells and to a less extent in MEFs, but were not detected in frog blastulae (Figure 5E and Table S1). Except for mono-methyl (K27 or K36) and di-methyl (K36) states, frog blastulae contain the lowest methylation levels for the H3 27–40 peptide of all the samples tested.

Overall, these results indicate that frog blastulae and murine ES cells share high H3K4me2/me3 levels, but the former are dramatically undermethylated at the H3 27–40 peptide. In fact, uncommitted frog cells have a 15-fold higher level of the unmodified state of this peptide than pluripotent GS-1 ES cells. Based on this, the polycomb repression complex 2 (PRC2) activity seems to have a very different impact on the epigenomes of frog embryos and *in vitro* cultured murine ES cells.

### Stage specific histone modification profiles

**Figure 6:**
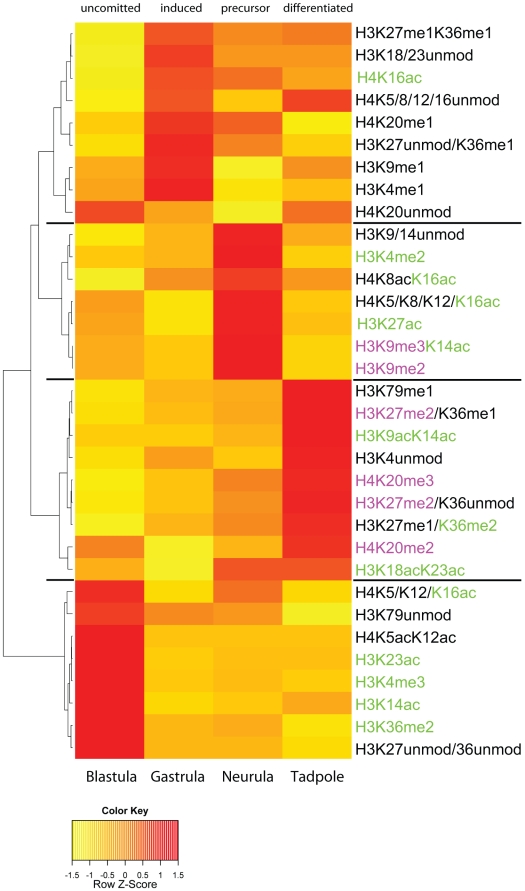
Stage-specific histone modification profiles. The heatmap visualizes clusters of histone modifications according to their relative abundance at the four developmental stages, which we have investigated. The hierarchical clustering analysis produces a dendrogram, shown on the left side, with four major branches that correspond to specific developmental stages. Modifications associated with transcriptional activity are highlighted in green, while modifications associated with repressed/silent states are shown in magenta. The four clusters define histone modifications profiles (HMPs), which reflect the gradual transition from uncomitted to determined cell fates.

We have observed significant quantitative changes in a relatively large number of histone modification states during frog embryogenesis. In order to investigate, whether particular developmental stages could be described by characteristic groups of histone modification states, we performed hierarchical clustering analysis to identify potential epigenetic patterns, and we then generated a Heatmap of the obtained clusters to visualize the results (Figure 6; see Materials and Methods for details). The dendrogram on the left axis of the Heatmap shows four well-defined clusters between the second and third level. The most obvious features of the heatmap are the four red-colored areas, each representing a cluster of histone modifications at their individual maximal abundance, which describe the four developmental stages that we analysed. The separation is very clear between blastula and tadpole clusters, where the red groups generally indicate a global shift from active to repressive histone marks as the embryonic cells undergo differentiation. It is less clear between gastrula and neurula stages, in particular at the bottom third of the heatmap, where several histone modification states persist at similar, intermediate abundances until the tadpole stage. As gastrula and neurula stages are biologically characterized by ongoing cellular diversification, these modifications may be associated with transitory features of germ-layer comitted precursor cell populations. Importantly, the apparent clustering provides unique evidence for a global, stepwise maturation of the embryonic epigenome in vertebrate embryos.

## Discussion

To our knowledge, this study represents the first description of bulk histone modifications and their steady state flux as it occurs *in vivo* during vertebrate embryogenesis. Using high accuracy LC-MS/MS and MALDI-TOF mass spectrometry, we have detected 59 different modification states on 18 sites - all Lysine residues, with the exception of histone H3 Serine 57 - derived from canonical H3 and H4 histones. These results provide key insight into global alterations of histone PTMs during development from uncomitted cells of the late blastula to the differentiated tissues of hatched, premetamorphic tadpoles.

Mass spectrometry is a very quantitative technique, as shown by the proportionate increase in XICs upon loading of different amounts of synthetic peptides (both unmodified and modified) over several orders of magnitude [56]. In addition, we have detected the full range of possible modifications for many peptides. The modifications that we have not found are known to be either of low abundance (Arginine methylation, Lysine ubiqitination) or unstable (phosphorylation; see [57], [58], [59]. Finally, Western blots for the H3K27me3 levels of frog embryos and ES cells confirm the quantitative differences obtained by mass spec. Altogether, this indicates that the reported relative abundances of modification states are robust, and that it is very unlikely that they could be inflated due to non-detected modifications.

At the time of germ layer induction, frog embryonic cells are characterized by a low point of repressive histone modifications, including H3K27me3. Our findings are in agreement with recent ChIP-seq results from Xenopus tropicalis [22] and suggest that the formation of somatic cell lineages in Xenopus species occurs from an epigenetic state practically devoid of bivalent domains. Future studies in Xenopus will have to address, whether the derepressed state at blastula exists also in early pre-MBT stages such as the morula (see also below). Global changes in histone modifications occur in mouse embryos between fertilisation and the blastocyst stage (reviewed in refs. [10], [11]. However, histone PTMs have not been investigated from post-implantation embryos, except for one pioneering study using carrier ChIP [19]. Here, both K4me3 and K27me3 marks were found on several developmental loci in epiblast tissue, but the bivalent status of these loci was not rigorously established and the opposing marks may derive from distinct cell populations. Nevertheless, this study demonstrates that K27me3 exists in the mouse embryo at the time of germ layer formation, unlike in Xenopus embryos.

By hierarchical clustering analysis, we found that a large fraction of the observed histone modifications fall into profiles that apparently characterize each of the analysed embryonic stages. This finding extends the hypothesis of individualized epigenomes for different cell types (e.g. [15], [20], [21], [60], [61]) to whole embryos. This surprising result does not necessarily imply that all cells of an embryo at a given stage share the same histone PTM profile, but rather reveals the existence of stage-specific constraints that shape the histone modifications according to global cellular transitions, such as the development from pluripotency to germ layer precursor to committed and differentiated cell states. In line with this hypothesis, the clearest differences are observed between histones from post-MBT, uncomitted cells of blastulae (NF9) and histones from the largely differentiated, somatic cells of tadpoles (NF37). Overall, the four clusters describe a general decrease in the abundance of active histone PTMs with a concomittant increase of repressive histone modifications. For future experiments, these clusters provide a blueprint of stage-specific chromatin states that can be used to investigate the impact and interrelationship of individual histone modifying enzymes.

We noted that some modification types - e.g. K9me1, K14ac, K36me2 on histone H3, or K16ac on histone H4 - remained remarkably constant (<two-fold changes) over this time course. Even though the local distribution of the PTMs at these histone residues will vary between cell types, this finding suggests relatively static or basic functions for them. For other sites, the steady state of PTMs can change very strongly within few hours, most notably between the late blastula and midgastrula stages. Here, the largest differences were observed on histone H3 for K27me1/K36me1 and K27me1/K36me2 (18-fold and 9.8-fold increase, respectively), as well as for acetylation and mono-methylation of H3K56 (11.5-fold and 18-fold decrease, respectively). Another example is represented by double-acetylation of Lysines 8 and 16 on H4, which rises from below detection limit to 11% abundance within this short time window. The disproportionate change in the steady state of these specific PTMs suggests that they are regulated by some developmental mechanism, possibly involving the signalling cascades that drive embryonic induction and cell differentiation.

Since Lysines 27 and 36 are present on the same tryptic peptide, it was possible to distinguish a total of 12 different modification states. Specifically, almost half of the unmodified fraction of this peptide becomes modified in steady state between blastula and gastrula. This dramatic increase in K27 methylation is consistent with the results of recent ChIP-studies in *X. tropicalis* and *D. rerio*, which reported a hierarchical acquisition of H3K4 and K27 methyl marks [22], [23]. In extension to these studies, however, we have found that the K27me3 mark is at least 100-fold less abundant in *Xenopus* early embryos than in cultured frog or mammalian cell lines, including murine ES-cells. As a consequence, the K27me3 density in chromatin may be rather low throughout the embryo. Alternatively, it could be high within a subfraction of either cells or genomic regions, and consequently being depleted from the rest. Whatever may be the case, this finding is very unexpected, given current models on the epigenetic nature of pluripotency. Functional tests are required to clarify, whether at this low abundance (<0.1%) H3K27me3 represses transcription in Xenopus blastulae as reported for human and murine ES cells, in which H3K27me3 is much more abundant (refs. [1], [15], [62]; and our data here).

It is not known, why the chromatin of the frog blastula is so devoid of the H3K27me2/me3 marks. Recently, an antagonistic relationship between methylation and acetylation of Lysine 27 has been reported that reflects a competition between PcG and trithorax/MLL complexes, which recruit H3K27 methyltransferase or acetyltransferase activities, respectively [63]. Since frog embryos contain the highest relative K27ac levels in our data set, one might speculate that some regulatory mechanism in blastula embryos favors MLL-mediated recruitment of HATs. However, a direct competition between trx and PcG proteins on target genes is unlikely to explain entirely the initial depletion in H3K27 methylation, because K27ac levels remain high during subsequent development despite the dramatic increase in K27me2/me3 from gastrula stages onwards.

Nicklay and colleagues have incubated sperm nuclei with Xenopus egg extract *in vitro* to generate chromatin of “early embryo equivalent” that may represent the chromatin state before or at MBT [21]. This sample contains quite abundant K27me3 levels (estimated 10–50%), confirming the maternal expression of biologically active PRC2 components in early embryos [64]. However, neither we (this study) nor others [22] have found evidence for this in NF9 blastulae. If chromatin of the “early embryo equivalent” corresponds to a physiological state, one would have to postulate that practically all K27me3 marks become erased before midblastula. The presence of maternal and potentially active PRC2 complexes in turn suggests for the unmanipulated frog embryo that PRC2 activity is either negatively controlled or efficiently antagonized by demethylation [65] around the onset of zygotic transcription. The sudden increase of K27me2 at gastrula suggests a regulatory switch that quite rapidly brings a significant portion of the epigenome under the influence of the *polycomb* system. This switch may include the upregulation of Suz12 mRNA levels that has been described during gastrulation [64].

During the amphibian blastula-gastrula transition, pluripotent embryonic cells are programmed by induction to germ layer related cell fates [28], [29]. This phase corresponds in mammalian embryos to the specification of the epiblast at implantation, from when on pluripotency is gradually lost [66]. Although being low abundant, acetylation of H3K56 is specifically enriched at promoters bound by the core pluripotency factors Oct4/Sox2/Nanog. When ES cells differentiate, K56ac relocates to developmental regulatory genes [45]. We have found that K56ac levels drop more than ten-fold between blastula and gastrula (Figure 2 and Table S1). It is possible that the transient depletion of K56ac from gastrula chromatin reflects the reported translocation of this mark, when pluripotency is lost in ES cells, and possibly in mammalian embryos. At the same time, the embryonic K9me1 level rises to a transient maximum of 20% abundance. H3K9me1 has been recognized as a stable mark of cis-regulatory elements for both active and inactive genes [1]. Together, the observed changes in the H3K56ac and H3K9me1 profiles may thus be important for the programming of cell fates.

The extraordinary abundance of H3K4me3 (almost 30%) in frog blastulae represents another puzzling observation. In mammals, the H3K4me3 mark is found on gene promoters and enhancers. Given that frog and human genomes are comparable in size and gene numbers, this level exceeds the expected genome proportion represented by these transcriptional regulatory elements. Notably, murine ES cells have similarly high levels, and even in somatic cells (A6, XTC and MEFs) its abundance is around 10% (see Figures 2, 5 and S3). Due to the unusual biophysical properties of this peptide, we could not record MS2 events, which would have allowed us to distinguish acetylated and trimethylated states. Although H3K4 acetylation levels are generally low [47], it is possible that some fraction of the H3K4me3 peak represents acetylated peptides. Nevertheless, one may speculate that H3K4me3 could be deposited differently in embryonic cells compared to somatic cells. In addition to the typical promoter-proximal peaks, which have been observed in recent genome-wide ChIP experiments both in Xenopus [15] and zebrafish [16], this mark could exist at higher base-levels throughout the embryonic epigenome, and/or be enriched significantly in still unannotated areas. The high abundance of H3 K4me3 in embryos certainly merits further investigation.

In general, the HMP of the frog blastula describes a highly permissive chromatin environment at the time of embryonic induction that is remarkably devoid of repressive methyl marks on H3K9, H3K27 and H4K20. This transcriptionally permissive epigenome may mechanistically explain, why some region or cell-type specific genes are initially activated in apparently ectopic manners in frog embryos [67], [68]. Subsequent refinement of gene expression patterns during gastrulation requires *de novo* synthesis of repressors [69], possibly including epigenetic repressors such as the PRC2 component Suz12 [64]. Based on our data, and on ChIP-Seq derived genome wide maps for H3K4 and H3K27 methyl marks [22], the epigenetic state of pluripotent Xenopus embryonic cells can thus be defined as “active” and “derepressed”. In this sense, they differ significantly from pluripotent mammalian ES cells and early post-implantation mouse embryos, which contain represssive marks such as H3K27me3 (our data on GS1-ES cells; [12], [19], [55] ). Our findings with Xenopus embryos underscore the necessity to further explore the epigenetic ground state of pluripotency in primary cells.

## Materials and Methods

### Ethics Statement

Animal work has been conducted in acordance with Deutsches Tierschutzgesetz; experimental use of Xenopus embryos has been licenced by the Government of Oberbayern (Projekt/AZ ROB: 211-2531.6-11/2001).

### Embryos and cell lines

Embryos were generated by in vitro fertilization with adult X.laevis animals (Nasco, *Xenopus* Express) as described [54]. Stable cell lines A6 (derived from adult frog kidney tissue) [70] and XTC-2 (derived from Xenopus tadpole carcass) [71] ) were grown in 75% DMEM supplemented with 10% FCS, 100 u/ml penicillin/ 100 ng/ml streptomycin at 26°C in a humidified atmosphere with 5% CO_2_.

Murine embryonic fibroblasts were isolated from ICR mice at day 13 of pregnancy and subsequently grown in DMEM, 10% FCS, 2 mM L-glutamine, 50 U/ml penicillin, 50 µg/ml streptomycin. For chromatin analysis, non inactivated MEFs at passage 2 were used. Murine GS-1 ES cells were grown on inactivated MEFs (3 hrs. 8 µg/ml mitomycin C) in high glucose Dulbecco's modified Eagle medium supplemented with 10% heat-inactivated ES-qualified fetal calf serum, 2 mM L-glutamine, 50 U/ml penicillin, 50 µg/ml streptomycin, 1x nonessential amino acids (all reagents GIBCO BRL, Germany) and 0.1 mM ß-mercaptoethanol (Sigma, Germany). For chromatin analysis, the ES cells were transferred to feeder free conditions on gelatinated plates for three passages to avoid contamination of probe material with MEF derived chromatin. During feeder-free culture, ES cells were kept undifferentiated by adding 1000 units/ml purified recombinant mouse leukemia inhibitory factor (ESGRO; Life Technologies, Inc., Grand Island, N.Y.) to the medium. Monolayers were passaged by trypsinization at 70–80% confluency and the cells were maintained at 37°C in a humidified atmosphere of 5% CO_2_/95% air.

### Histone Extraction

Around 50 to 100 embryos developed to Blastula (NF9, 7 hpf), Gastrula (NF12, 13 hpf), Neurula (NF18, 20 hpf), and Tadpole (NF37, 54 hpf) stages were harvested and washed with 110 mM KCl, 50 mM Tris/HCl (pH 7.4 at 23°C), 5 mM MgCl_2_, 0.1 mM spermine, 0.1 mM EDTA, 2 mM DTT, 0.4 mM PMSF, 10 mM Na-butyrate. Nuclei of the embryos were prepared by centrifugation with 2600xg, 10 min (3–18, Sigma) after homogenization by a 5 ml glass-glass douncer (Braun, Melsungen). For cell lines, 10^7^ cells were collected and washed twice with 1 ml PBS, 0.1 mM spermine, 0.1 mM EDTA, 2 mM DTT, 0.4 mM PMSF, 10 mM Na-butyrate and then resuspended in 1 ml PBS buffer containing 0.3% Triton. The cells were put on a rotating wheel at 4°C for 20 min and then centrifuged with an Eppendorf Centrifuge 5417C with top speed. The supernatent containing the cytoplasmatic fraction was discarded. The nuclear pellets of both *Xenopus* embryos and cell lines were resuspended in 1 ml 0.4 M HCl, incubated on a rotating wheel over night and dialysed against 3l of 0.1 M acetic acid/1 mM DTT. The dialysed histone solution was vacuum-dried in a Concentrator Plus (Eppendorf) and stored at −20°C.

### Mass Spectrometry

Histones were separated by sodium dodecyl sulfate-polyacrylamide gel electrophoresis (SDS-PAGE). Coomassie blue-stained bands were excised and propionylated as described before [34]. Digestions were carried out overnight with 200 ng sequencing grade trypsin (Promega); Before analysis, the peptide solution was desalted using Zip-Tips (Millipore) or porous carbon material (TipTop Carbon, Glygen). Bound pepties were eluted in matrix solution (saturated -cyanohydroxycinnamic- acid [Sigma] dissolved in 50% acetonitril [vol/vol], 0.3% trifluoroacetic acid [vol/vol]) and spotted onto a target plate. The target plate was loaded in a Voyager DE STR spectrometer (Applied Biosystems). The resulting spectra were analyzed using the Data Explorer and in-house-developed software Manuelito (open source: http://sourceforge.net/projects/manuelito ). For quantification of the different post-translational modifications of the various peptides, the relative intensities of each were measured. The areas of all modifications of one peptide were summarized and thus the percentage of each modification was calculated. The statistical significance of quantitative changes during development or between samples was determined by Student's T-test.

For LC-MS/MS analysis the digested histones were loaded onto a reversed-phase HPLC in an analytical C18 column (75 µm i.d packed with C18 PepMap™, 3 µm, 100Å by LC Packings) and eluted using a 80 min gradient from 5 to 60% ACN in 0.1% FA. Spectra were recorded by a LTQ-Orbitrap mass spectrometer operating in a top six mode where the six most intense peptide ions with charge states between 2 and 4 were sequentially isolated and fragmented in the linear ion trap by collision-induced dissociation (CID). All fragment ion spectra were recorded in the LTQ part of the instrument. For all measurements with the orbitrap detector, 3 lock-mass ions from ambient air (m/z = 371.10123, 445.12002, 519.13882) were used for internal calibration as described. Typical mass spectrometric conditions were: spray voltage, 1.5 kV; no sheath and auxiliary gas flow; heated capillary temperature 200°C. For the analysis of different histone modifications the resulting RAW files were converted into DTA and searched against the NCBI non-redundant database using the SEQUEST search algorithm to identify the corresponding histones using the BIOWORKS3.3 software package (Thermo). In order to determine the posttranslational modifications on the histone peptides, the data files were searched against a targeted histone database containing *Xenopus laevis* histone molecules from the NCBI non-redundant database by SEQUEST search algorithm with the following modification settings (static modification on lysine: 56.0262; variable modifications: me1: 14.01565; me2: -27.99490; me3: -13.97925; ac: -14.10565 on K and phos: 79.96633 on S). The resulting SEQUEST files were filtered for a peptide probability score of 0.0005 and Xcorrelation values of 1.5, 2.0, 2.5 and 3 for charge states 1, 2, 3 and 4 accordingly. A similar search against a reversed and scrambled histone decoy database did not lead to an identification of peptides that matched the filter criteria. Therefore we estimated the false discovery rate to be less than 1%. For quantification, only modifications with a probability score of lower than 0.0005 and top hits were used. For all peptides, which were quantified, an extracted ion chromatogram (XIC) was obtained from the raw file using the Xcalibur 2.0.7 software using the default ICIS peak detection algorithm (Thermo), extracting doubly and triply charged ions with a user defined mass tolerance of 5–10 ppm and a mass precision of 4 decimals to be able to distinguish between nominally isobaric modifications such as acteylation or trimethylation. The retention time for each specific peptide on the chromatography was highly specific when we used the same LC MS/MS method. We therefore only used these XIC areas for quantification, which eluted at time points where we observed at least two MS/MS spectra that unambiguously identified the identity of the peptide and the position of the modification within the peptide.

### Quantitative Western blotting

Western blot analysis for H4 K20me3 was carried out according to standard protocols [72]. Nuclei of 0.5–2 embryo equivalents were used, depending on developmental stage. After SDS-PAGE, proteins were blotted with 1.5 mA/cm2 for 1 h to nitrocellulose membranes (porcello NCL, Macherey-Nagel), which were blocked for 1 h using PBS, 3% BSA. Primary antibodies detecting H3K27me3 (1∶3 000; ref. [73]), H4K20me3 (1∶500; ref. [73]) or core region-H3 (Abcam, 1∶25.000) were incubated using PBS, 3% BSA. Secondary fluorophore linked antibody (Li-cor, IRDye 700DX goat anti-rabbit, 1∶5 000) was incubated in PBS, 5% Milk powder, 0.1% Tween 20. Fluorescent signals were recorded using Li-cor Odysse wet membrane technique for 700 nm absorbance.

### Heatmap Generation

We generated the Heatmap shown in Figure 6 using R (http://www.r-project.org) and the *gplots* package (http://CRAN.R-project.org/package=gplots). All functions were called using default parameters if not indicated otherwise. We first calculated the mean and the standard deviation of the quantifications obtained by Mass Spectometry across the four developmental cell stages (each stage containing two biological replicates) for each of the 57 modifications. Histones with small mean values contain low Mass Spectometry quantifications that could obscure the clustering analysis that we observe in the heatmap. Thus, as a preprocessing step of the data, we discarded modifications with mean values less than 0.5% (22 modifications). In order to compare appropriately the contribution of each modification to the specific cell stages, we standardized the mass spectometry quantifications individually using Z-scores. Using these Z-scores we then produced the Heatmap using the Ward's minimum variance method to perform the hierarchical clustering analysis.

## Supporting Information

Figure S1 **Conservation of nucleosomal core histone sequences.** ClustalW alignment of core nucleosomal histone proteins from Xenopus, human, mouse and Drosophila. Genbank accession numbers of *Xenopus* proteins – H3.2 (AAA49770), H3.3 (NP_001080065), H4 (AAA49771).
(EPS)

Figure S2 **Histone modification sites and sequence coverage of Xenopus histones H3 and H4 after tryptic digest.** Underlined are the amino acid sequences analyzed here by Mass Spectrometry. Amino acid residues known to be covalently modified in mammalian histones (see Bhaumik et al., 2010) are 100% conserved in frog histones and shown in bold letters. Histone H3.2 and H3.3 sequences differ in few positions, one of which (position 30 - Serin in H3.3 versus Ala in H3.2) is present on the peptide H3 27–40. Mass spec data refers to histone H3.2, which is much more abundant than H3.3.
(EPS)

Figure S3 **Histone modification patterns of Xenopus somatic cell lines.** Bar-Charts displaying the relative abundance of histone modifications from *Xenopus* somatic cell lines A6 and XTC-2 (see color code in panel **F**). Panels **A**–**D, G** and **I**–**K** show data from Orbi-Trap Mass Spectrometry; error bars indicate SD of two independent biological replicates. Panels **E** and **H** contain MALDI-TOF data; error bars indicate SD of three independent biological replicates. Abbr.: unmod  =  unmodified peptide, Kac  =  acetylated lysine residue, Kme1  =  mono-methylated, Kme2  =  di-methylated, Kme3  =  tri-methylated lysine residues. Where applicable, p values are given by numbers above brackets to indicate significant differences in histone modifications between cell types.
(EPS)

Figure S4 **H4K20me3 levels in cultured mammalian and frog cells.** Immunoblotting for total histone H3 and histone H4 trimethylated at lysine 20. Upper panel – western blot Odyssey infrared imaging signals; lower panel – bar chart showing the abundance of H4K20me3 levels relative to total histone H3 protein in murine GS-1 ES cells, MEFs and Xenopus cell lines A6 and XTC. Error bars (SD).
(EPS)

Figure S5 **Quantification of H3.3/H3.2 ratios in Xenopus bulk histones isolated from different developmental stages**. The ratios of MS/MS events for the unique H3.3 peptide KSAPSTGGVKKPHR and the corresponding H3.2 peptide KSAPATGGVKKPHR were calculated and used as a proxy to determine the ratio of H3.3/H3.2 in histones isolated from various embryo samples. Abbreviations: NF9  = Blastula, NF12  =  Gastrula, NF18  =  Neurula, NF37  =  Tadpole, A6 - frog kidney cell line. Values represent the mean of at least two independent biological replicates. Error bars represent the calculated SD values.
(EPS)

Figure S6 **Distinct histone modification patterns reveal adequate separation of murine ES cells from MEF-feeder cells.** Bar-Charts for acetylated histones H4 4–17 (**A**) and H3 18–26 (**B**) peptides. Data from Orbi-Trap Mass Spectrometry, error bars indicate SD of two independent biological replicates. Numbers above brackets provide p-values for quantitative differences between ES and feeder cells. **C**) Murine GS-1 ES cells cultured on γ-irradiated MEF feeder cell layer. **D**) GS-1 ES cells after two passages with recombinant LIF-supplemented medium without feeder cells.
(EPS)

Table S1 **Tandem Mass Spectrometry - quantitative results of histone H3 modifications.** Indicated is the peptide with its amino acid sequence, its different modifications and their masses (M(cal)H+), and the percentile value of the modification according to the LC quantification. Abbreviations: NF9  =  Blastula stage, NF12  =  Gastrula stage, NF18  =  Neurula stage, NF37  =  Tadpole stage, A6 and XTC-2 are stable *X.laevis* cell lines, ES: murine GS-1 ES cell line, MEFs  =  murine embryo fibroblasts. M(cal)H+ is the mass of the positively charged peptide, Av. RT is the average Retention time of the peptide on the C18 micro colum of the reversed-phase LC-MS. Values represent the mean of two independent biological replicates.
(EPS)

Table S2 **Tandem Mass Spectrometry - quantitative results of histone H4 modifications.** Indicated is the eptide with its amino acid sequence and the different modifications and their masses (M(cal)H+) and the percentile value of the modification according to the LC quantification. Abbr.: NF9  =  Blastula stage, NF12  =  Gastrula stage, NF18  =  Neurula stage, NF37  =  Tadpole stage, A6 and XTC are stable *X.laevis* cell lines, ES: murine GS-1 ES cell line, MEFs  =  murine embryo fibroblasts. M(cal)H+ is the mass of the positively charged peptide, Av. RT is the average Retention time of the peptide on the C18 micro colum of the reversed-phase LC-MS. Values represent the mean of two independent biological replicates.
(EPS)

Table S3 **Tandem Mass Spectrometric MS2 events after Collision Induced Dissociation (CID) of Histone H3.** Indicated is the peptide with its amino acid sequence and the different modifications and their masses (M(cal)H+) and the number of MS2 events for each modification. Numbers highlighted in grey are not used for quantification as their peptide probability score was lower than other modifications of the same modification state. Abbreviations: NF9  =  Blastula stage, NF12  =  Gastrula stage, NF18  =  Neurula stage, NF37  =  Tadpole stage. A6 and XTC are stable *X.laevis* cell lines; ES: murine GS-1 ES cell line, MEFs  =  murine embryo fibroblasts. “M(cal)H+” is the mass of the positively charged peptide. “Av DeltaM” lists the average mass deviation of the theoretical mass and the measured values. “Av RT” describes the average retention time of the peptide on the C18 micro-column of the reversed-phase LC-MS. “z” is the charge of the most abundant peptide. Values represent the mean of two independent biological replicates.
(EPS)

Table S4 **Tandem Mass Spectrometric MS2 Events after Collision Induced Dissociation (CID) of Histone H4.** Indicated is the peptide with its amino acid sequence and the different modifications and their masses (M(cal)H+) and the number of MS2 events for each modification. Numbers highlighted in grey are not used for quantifications as their peptide probability score was lower than other modifications of the same modification state. Abbreviations: NF9  =  Blastula stage, NF12  =  Gastrula stage, NF18  =  Neurula stage, NF37  =  Tadpole stage, A6 and XTC-2 are stable *X.laevis* cell lines, ES: murine GS-1 ES cell line, MEFs  =  murine embryo fibroblasts. “M(cal)H+” is the mass of the positively charged peptide. “Av DeltaM” lists the average mass deviation of the theoretical mass and the measured values. “Av RT” describes the average retention time of the peptide on the C18 micro-column of the reversed-phase LC-MS. “z” is the charge of the most abundant peptide. Values represent the mean of two independent biological replicates.
(EPS)
